# Supplementary material for: An Integrative Review of Recovery Services to Improve the Lives of Adults Living with Severe Mental Illness
Source: Int J Environ Res Public Health. 2021 Aug 23;18(16):8873. doi: 10.3390/ijerph18168873 (PMC8393579; doi:10.3390/ijerph18168873)
Supplement: Supplementary file 1 [file ijerph-18-08873-s001.zip › ijerph-1263766-supplementary.pdf]

International Journal of
Environmental Research and Public Health

MDPI

Table S1. Data extraction forms.

**Study ID**	
**Study Details**	
Citation	
Year of publication(s)	
Author(s)	
Contact details of lead author	
Funder / sponsoring organisation	
Publication type<br>Example:<br>• Journal article<br>• Report (specify)<br>• Case study<br>• Other	Journal article
**Publication Source**	
Methodology (if applicable)<br>• Study design	
• Type of data	
• Data collection	
• Sampling	
• Data analysis	
• Participants/No. of studies included	
Age range	
Sex	
Study setting	
The objective of the study	
Study type<br>Please specify the focus of the paper?<br>• Recovery<br>• Disability support	
Sector<br>The paper may focus on one or more sectors, for example,<br>• Occupational therapy<br>• Recreational activities<br>• Social care<br>• Treatment	
**Disability support**	**Please identify the model of disability support services for adult with mental illness?**
**Interventions**	**Please identify any disability support interventions implemented for adult with mental illness?**
**Period of project implementation**	**Please state the period (months/years) of implementing the intervention**
**Outcome of intervention**	**Please state the outcome of the disability support intervention for adult with severe mental illness?**
**Concept of Recovery**	**Please specify the concept of recovery in treating adult with severe mental illness?**

**Please describe the role of recovery in the social inclusion and community acceptance of adult with severe mental illness?**	
**Recovery intervention**	**Please identify any recovery interventions implemented for adult with mental illness?**
**Period of project implementation**	**Please state the period (months/years) of implementing the intervention**
**Outcome of intervention**	**Please state the outcome of the recovery intervention for adult with severe mental illness?**
**Please report on any additional information on disability support and recovery services**	
**Recommendation**	
**Identifiable references to follow up**	

Table S2. Methodological Quality assessment Criteria.

**Reviewer 1**				
**Reviewer 2**				
**Author (s)**				
**Methods**				
**Study design**				
**Data**				
**Sampling**				
**Analysis**				
**Types of Study**	**Methodological Quality assessment Criteria**	**Yes**	**No**	**Cant tell**
**Screening Questions (for all types)**	Are there clear research questions or objectives?	☐	☐	☐
	Do the collected data address the research question?	☐	☐	☐
	Further appraisal is not feasible when the answer is 'No' or 'Can't tell' to one or both screening questions			
**Qualitative**	1.1 Is there congruity between the stated philosophical perspective and the research methodology?	☐	☐	☐
	1.2 Are the sources of qualitative data (archives, documents, informants, observations) relevant to address the research question?	☐	☐	☐
	1.3 Is the process for analysing qualitative data relevant to address the research question?	☐	☐	☐
	1.4 Are participants, and their voices, adequately represented? (adequate quotes and text been used to represent the concept discussed)	☐	☐	☐
	1.5 Is there a statement locating the researcher culturally or theoretically? (Are the beliefs and values, and their potential influence on the study declared?)	☐	☐	☐
	1.6. Is the influence of the researcher on the research, and vice-versa, addressed? *(Addressing the potential for the researcher to either influence or to be influenced by the study)*	☐	☐	☐

	1.7. Do the conclusions drawn in the research report flow from the analysis, or interpretation, of the data?	☐	☐	☐
	1.8. Is the ethical issues adequately addressed? *(statement indicating appropriate ethics approval)*	☐	☐	☐
**Quantitative randomized controlled (trials)**	2.1. Is there a clear description of the randomization (or an appropriate sequence generation)?	☐	☐	☐
	2.2. Is there a clear description of the allocation concealment or blinding when applicable)?	☐	☐	☐
	2.3. Are there complete outcome data (80% or above)?	☐	☐	☐
	2.4. Is there low withdrawal/drop-out (below 20%)?	☐	☐	☐
	2.5 Are the groups comparable at baseline?	☐	☐	☐
	2.6 Are there complete outcome data?	☐	☐	☐
	2.7 Are outcome assessors blinded to the intervention provided?	☐	☐	☐
	2.8 Did the participants adhere to the assigned intervention?	☐	☐	☐
**Quantitative non-randomized (Cohort study, case-control study, analytical cross-sectional)**	3.1. Are participants recruited in a way that minimizes selection bias?	☐	☐	☐
	3.2 Were the criteria for inclusion in the sample clearly defined?	☐	☐	☐
	3.3 Were the study subjects and the setting described in detail?	☐	☐	☐
	3.4 Were objective, standard criteria used for measurement of the condition?	☐	☐	☐
	3.5 Were the outcomes measured in a valid and reliable way?	☐	☐	☐
	3.6 Was appropriate statistical analysis used?	☐	☐	☐
	3.7 Is the ethical issues adequately addressed? (statement indicating appropriate ethics approval)	☐	☐	☐
	3.8 Do the conclusions drawn in the research report flow from the analysis, or interpretation, of the data?	☐	☐	☐
	3.9 Are measurements appropriate (clear origin, or validity known, or standard instrument; and absence of contamination between groups when appropriate) regarding the exposure/intervention and outcomes?	☐	☐	☐
	3.11 In the groups being compared (exposed vs. non-exposed; with intervention vs. without; cases vs. controls), are the participants comparable, or do researchers take into account (control for) the difference between these groups?	☐	☒	☐
	3.12 Are there complete outcome data (80% or above), and, when applicable, an acceptable response rate (60% or above), or an acceptable follow-up rate for cohort studies (depending on the duration of follow-up)?	☐	☐	☐
**Quantitative descriptive**	4.1. Is the sampling strategy relevant to address the quantitative research question (quantitative aspect of the mixed methods question)?	☐	☐	☐
	4.2. Is the sample representative of the population under-study?	☐	☐	☐
	4.3. Are measurements appropriate (clear origin, or validity known, or standard instrument)?	☐	☐	☐
	4.4. Is there an acceptable response rate (60% or above)?	☐	☐	☐
**Mixed methods**	5.1. Is the mixed methods research design relevant to address the qualitative and quantitative research questions, or the qualitative and quantitative aspects of the mixed methods question?	☐	☐	☐
	5.2. Is the integration of qualitative and quantitative data (or results relevant to address the research question?	☐	☐	☐
	5.3 Are the outputs of the integration of qualitative and quan-	☐	☐	☐

	titative components adequately interpreted?			
	5.4 Are divergences and inconsistencies between quantitative and qualitative results adequately addressed?	☐	☐	☐
	5.5 Do the different components of the study adhere to the quality criteria of each tradition of the methods involved?	☐	☐	☐
	5.6 Is appropriate consideration given to the limitations associated with this integration, e.g., the divergence of qualitative and quantitative data in a triangulation design?	☐	☐	☐
	Apply the criteria use for qualitative data for the qualitative component and quantitative component respectively.			
**Overall Quality Score**	*Comments on score: The overall quality score for the paper is…. % and so meet the criteria for assigning high quality*	☐ Low (25%)<br>☐ Medium (50%)<br>☐ High 70% - 100%		
**Reviewer 2**	*Comments:*			

NB: Scoring metrics. *The score can be computed by counting the total number of "yes" and expressing them as a percentage ie below 25% represent Low Quality, 50% represent Medium Quality, and 70% and above represent high Quality.*
